# Genomics and tumor microenvironment of breast mucoepidermoid carcinoma based on whole-exome and RNA sequencing

**DOI:** 10.1186/s13000-024-01439-8

**Published:** 2024-01-19

**Authors:** Yan Ge, Xingtao Lin, Jiao He, Wendan Chen, Danyi Lin, Yihong Zheng, Lingling Yang, Fangping Xu, Zhi Li

**Affiliations:** 1 Department of Pathology, Guangdong Provincial People’s Hospital/Guangdong Academy of Medical Sciences, Southern Medical University, Guangzhou, China; 2 Department of Pathology, Guangdong Provincial People’ s Hospital Ganzhou Hospital (Ganzhou Municipal Hospital), 49 Dagong Road, Zhanggong District, Ganzhou, China; 3 Geneseeq Research Institute, Geneseeq Technology Inc., Nanjing, China

**Keywords:** Breast, Mucoepidermoid carcinoma, Whole-exome sequencing, RNA-sequencing

## Abstract

Mammary mucoepidermoid carcinoma (MEC) is a rare entity. The molecular characteristics of breast MEC have not been fully investigated due to its rarity. We performed a retrospective study among 1000 patients with breast carcinomas and identified four cases of breast MEC. Clinical and demographic data were collected. Immunohistochemistry panels which were used to diagnose salivary gland MEC and breast carcinomas were also performed. *MAML2* rearrangements were detected by FISH and fusion partners were identified by RNA sequencing. Whole-exome sequencing (WES) was used to reveal the genomes of these four breast MEC. Then, the biological functions and features of breast MEC were further compared with those of invasive breast carcinomas and salivary gland MEC.

According to Ellis and Auclair’s methods, these four breast MEC could be classified as low-grade breast MEC. All the patients were alive, and disease-free survival (PFS) ranged from 20 months to 67 months. Among these four breast MEC, two cases were triple-negative, and the other two cases were found to be ER positive, with one also showing HER2 equivocal by immunohistochemical staining, but no amplification in FISH. FISH analysis confirmed the presence of the *MAML2* translocation in three of four tumors, and *CRTC1-MAML2* fusion was confirmed in two of them by RNA-sequencing. The average coverage size of WES for the tumor mutation burden estimation was 32 Mb. *MUC4, RP1L1* and *QRICH2* mutations were identified in at least three tumors, and these mutation also existed in breast invasive carcinoma databases (TCGA, Cell 2015; TCGA, Nature 2012). The results showed that there were many genes in breast MEC overlapping with the breast invasive carcinoma databases mentioned above, range from 5 to 63 genes (median:21 genes). Next, we assessed immune cell infiltration levels in these tumors. In all these tumors, M2 macrophages and plasma cell were in the high infiltration group. Our breast MEC showed different results from the salivary gland MEC, whose plasma cells were in the low infiltration group. Overall, we first analyzed the genomics and tumor microenvironment of breast mucoepidermoid carcinoma and proposed our hypothesis that although MECs arising in the breast resemble their salivary gland counterparts phenotypically, our findings indicate that breast MECs probably resemble invasive breast carcinomas at the genetic level and immune cell infiltration levels. More cases and in deep research need to be done to further understand this rare carcinoma.

**Supplementary Information:**

The online version contains supplementary material available at 10.1186/s13000-024-01439-8.

## Introduction

Mucoepidermoid carcinoma (MEC) of the breast is a rare entity, of which the incidence is 0.2–0.3% of all mammary carcinomas [1]. To date, no more than 55 cases of breast MECs have been reported worldwide, mostly as single case reports [2]. Breast MECs share similar morphologic features with salivary counterparts. According to Ellis and Auclair’s methods, [3] which are the most commonly used grading systems for MEC in the salivary gland, breast MEC can also be separated into low-grade, intermediate-grade, or high-grade MEC. Immunohistochemistry assists in the diagnosis of MEC, as each cell type has a distinctive profile. Most reported breast MECs show triple-negative phenotypes. However, a recent hormonal receptor expression analysis yielded a conflicting result, in which some breast MEC samples were found to be ER positive [4]. MECs arising in the salivary gland and lung have been shown to harbor the t (11, 19) (q14–21; p12–13) translocation, which resulted in the *CRTC1-MAML2* fusion gene [5]. Bean GR [1] et al. were the first to demonstrate the presence of the *CRTC1-MAML2* fusion in breast MEC, which was later confirmed by other research groups [5, 6]. Nevertheless, the molecular characteristics of breast MEC have not been fully investigated due to its rarity.

In this study, we systematically investigated the genomic profiles of four low-grade breast MEC using whole-exome sequencing and RNA sequencing. Uncovering the gene mutation spectrum and molecular profile may shed light on the tumorigenesis of breast MEC.

## Materials and methods

A retrospective study was performed among 1000 breast carcinomas from 2009 to 2021 collected from the Department of Pathology, Guangdong Provincial People’s Hospital. Four breast MEC were identified. Clinical data, including age, sex, primary site, lymph node involvement, pathological findings, treatment strategies, clinical outcomes and follow up information, were collected from electronic medical records. The study was conducted in accordance with the principles in the Declaration of Helsinki, 2013. Approval for this study was obtained from the Guangdong Provincial People’s Hospital (KY-Z-2021-439-01).

Immunohistochemical studies were carried out with a panel of monoclonal and polyclonal antibodies reactive in formalin-fixed paraffin-embedded tissue sections using a peroxidase-labeled detection system, standard antigen retrieval protocols, and an automated immunostainer (BenchMark Ultra, Roche, Switzerland). The following antibodies were used: CK7 (OV-TL12/30, dilution 1:3200; Gene Tech, Shanghai, China), CK5/6 (EP24&EP67, dilution 1:100, Gene Tech, Shanghai, China), Ki-67 (MIB-1, dilution 1:30; BioGenex, Fremont, CA, USA), ER (SP1, dilution 1:1; Roche, Switzerland), PR (1E2, dilution 1:1; Basel, Roche, Switzerland) and c-erb-B2 (4b5, dilution 1:500; Ventana, South Dakota,USA), SMA (1A4, dilution 1:1600; Gene Tech, Shanghai, China), Desmin (D33, dilution 1:200; Gene Tech, Shanghai, China), P63 (4A4, dilution 1:1000; Gene Tech, Shanghai, China), CD34 (QBEnd10, dilution 1:800; Gene Tech, Shanghai, China), MUC4 (8G7, dilution 1:100, Gene Tech, Shanghai, China), CK14 (EP61, dilution 1:800, ZSGB-BIO, Wuxi, China), Calponin (CALP, dilution 1:3000,Gene Tech, Shanghai, China).

FISH was performed on 4-mm tissue sections with two colored split-apart probes for *MAML2* (Z-2014-200; ZytoVision, Bremerhaven, Germany), as previously described [7]. The green fluorochrome direct labeled probe hybridizes distal to the *MAML2* gene, and the orange fluorochrome directly labeled probe hybridizes proximal to that gene. Cells with two fusion signals of one orange and one green fluorochrome were scored as normal. Cells with rearrangements for the *MAML2* gene had one normal fusion signal, one orange and one green signal at a distance from each other. In each case, 100 cells were analyzed in the targeted region. A case was considered positive for *MAML2* rearrangement if a break-apart signal was identified in ≥20% of tumor nuclei.

RNA sequencing was performed on all four cases following a previously described protocol [7]. The relative infiltration level of 15 types of immune cell was estimated for each sample with CIBERSORT using gene expression data (transcripts per million) from RNA sequencing. The CIBERSORT package and gene expression signature matrix of 15 types of immune cells were downloaded from the web portal (http://cibersort.stanford.edu/) and ran locally.

WES sequencing was performed following protocols described previously [8]. The average sequencing depth was 150× for tumors and 60× for normal tissue controls. The average coverage size of WES for the tumor mutation burden estimation was 32 Mb. Mutation screening and definition were performed following protocols described previously [9]. FACETS were used to calculate gene-level and segment-level copy number variations (CNVs). If more than 60% of its segments had a consistent level of copy number alteration in one chromosome, this event was chromosomal instead of focal CNV events. For focal CNV events, deep amplifications and deep deletions were counted for further analyses. CNV events were used to calculate the chromosomal instability score, which was defined as the proportion of the length of the genome with segmented copy number alterations. Venn diagrams (https://bioinfogp.cnb.csic.es/tools/venny/) were constructed by comparing our data with public data (http://www.cbioportal.org/). CNVs were examined in invasive carcinomas of the breast, metaplastic carcinomas and mucoepidermoid carcinomas of the salivary gland. We also conducted immune cell infiltration estimate.

## Results

### Epidemiology

We reviewed 1000 breast carcinomas and identified four breast MEC. The clinicopathological findings are summarized in Table 1. All the patients were alive without evidence of disease progression in a period ranging from 20 months to 67 months (median follow up 40.5 months).

**Table 1:**
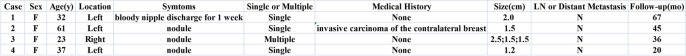
Clinical findings of the four reported cases

*F* female, *LN* lymph node metastasis at the time of primary diagnosis

### Morphology and IHC data

**Fig. 1:**
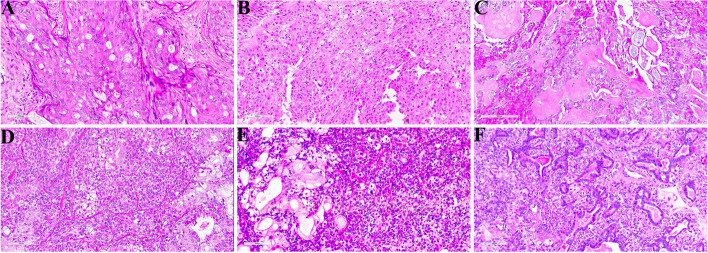
Morphologic features of breast mucoepidermoid carcinomas. **A** and **B** Epidermoid and intermediate cells were the major cell types found in case one and case three, mixing with a few mucinous cells and basaloid cells. **C** Case four was mainly composed of epidermoid and mucoid cells, with few basaloid and intermediate cells, and occasional intercellular bridges. **D** and **E** Case two was predominantly composed of basaloid cells, with different proportions of the other three types of cells. **F** Irregular adenoid structures could also be occationally observed in case two

The largest diameter of the tumors ranged from 1.2 to 2.5 cm, with an average of 1.5 cm. Under gross observation, three cases presented as single or multiple poorly circumscribed, irregular, nodular and cystic masses, while one case (case 2) was a solid nodule which had a well-circumscribed boundary. All cases were gray–yellow and fleshy with a firm texture. Similar to its salivary counterpart, the breast MEC was composed of different proportions of basaloid cells, intermediate (clear) cells, and epidermoid and mucinous cells (Fig. 1). Mitoses were infrequent in all 4 cases [1–3/10 high-power field (HPF)]. Neither necrosis nor true keratinization with squamous pearls was observed in these four tumors. Lymphovascular invasion was not identified in these cases.

**Fig. 2:**
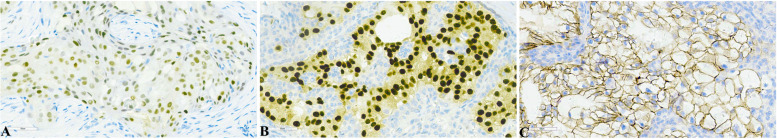
Microphotographs of the immunohistochemistry (IHC) in mucoepidermoid carcinoma of the breast. **A** About 60% of tumor cells were estrogen receptor (ER)- positive in case one. **B** About 40% of tumor cells were estrogen receptors (ER) -positive in case two. **C** The tumor cells in case showed HER2 equivocal

Various cell populations and their distribution of breast MECs could be highlighted by immunohistochemistry panel that was used in its salivary gland counterpart. Basaloid cells were CK14 and p63 positive. Intermediate cells expressed both p63 and HMWCK (CK5/6) but not LMWCK (CK7). Epidermoid cells responded to both HMWCK (CK5/6) and LMWCK (CK7). Mucinous cells preferentially reacted with LMWCK (CK7). Clearly, the peripheral p63 staining of intermediate cells could hardly be distinguished from myoepithelial cell staining; therefore we could not interpret ductal carcinoma in situ (DCIS) or invasive components solely relying on the p63 expression. Therefore, other myoepithelial cell markers, including calponin and SMA, were recruited, as all four types of cells were negative for SMA and calponin. In contrast to the epithelial markers mentioned above, the expression of hormone antibodies was also different in these four cases. Case three and case four were triple-negative breast carcinomas (Table 2). However, 60 and 40% of the tumor cells were estrogen receptors (ERs)-positive in case one (2+) and case two (3+), respectively (Fig. 2). At the same time, immunohistochemical examination confirmed that the tumor cells in case two showed HER2 equivocal (Fig. 2), but FISH examination showed no detectable HER2 amplification. Ki-67 staining showed low proliferation (less than 5%) in three cases. Nevertheless, case four showed a slightly higher Ki-67 index, approximately 10%.

**Table 2:**
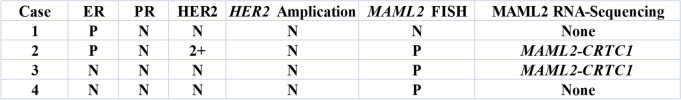
Immunohistochemical findings of hormone markers and expression of *HER2* and *MAML2* in the four reported cases

*P* positive, *N* negative, *ER* estrogen receptor, *PR* progesterone receptor, *HER-2* human epidermal growth factor receptor 2, *MAML2* mastermind like transcriptional coactivator 2, *CRTC1* CREB-regulated transcriptional coactivator 1

### Literature review

**Fig. 3:**
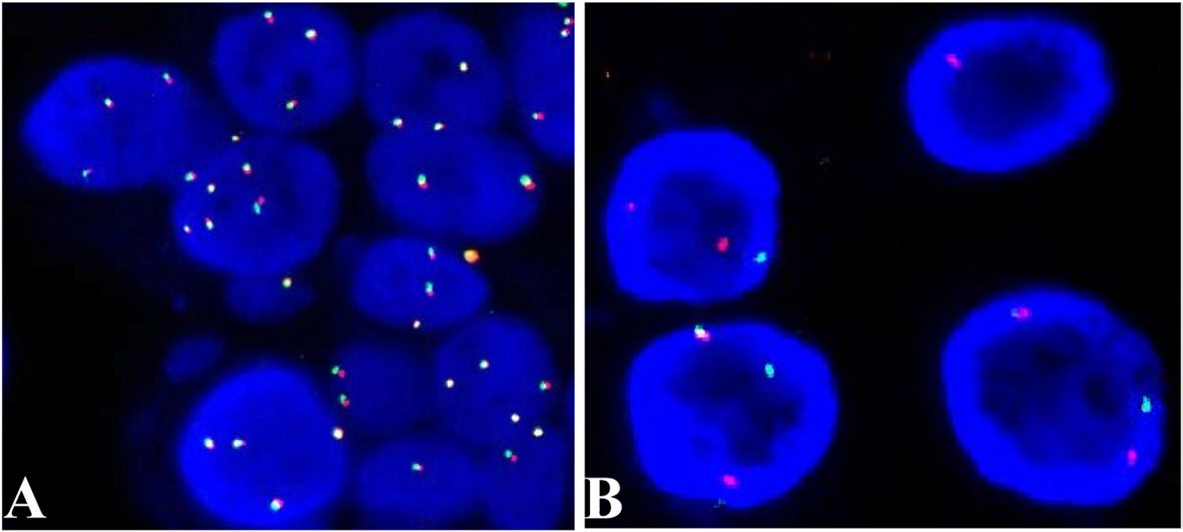
Fluorescence in situ hybridization analysis of *MAML2* (11q21) gene in mucoepidermoid carcinoma of the breast. The presence or absence of MAML2 translocation was determined by FISH using a dual-color, break apart probe. Cell nuclei were counterstained with DAPI (blue). **A** Representative images of cells (case one) without translocation. Each cell had two intact yellow signals. **B** Representative images of cells harboring the translocation. Each cell demonstrated one separate orange and one separate green signal

**Fig. 4:**
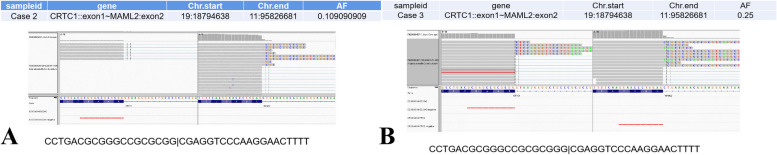
Schematic diagrams of *CRTC1-MAML2* fusion transcripts in our cohort. **A** *CRTC1-MAML2* rearrangement between the *CRTC1* exon 1 and *MAML2* exon 2 genes in case two. **B** *CRTC1-MAML2* rearrangement between the *CRTC1* exon 1 and *MAML2* exon 2 genes in case three

**Fig. 5:**
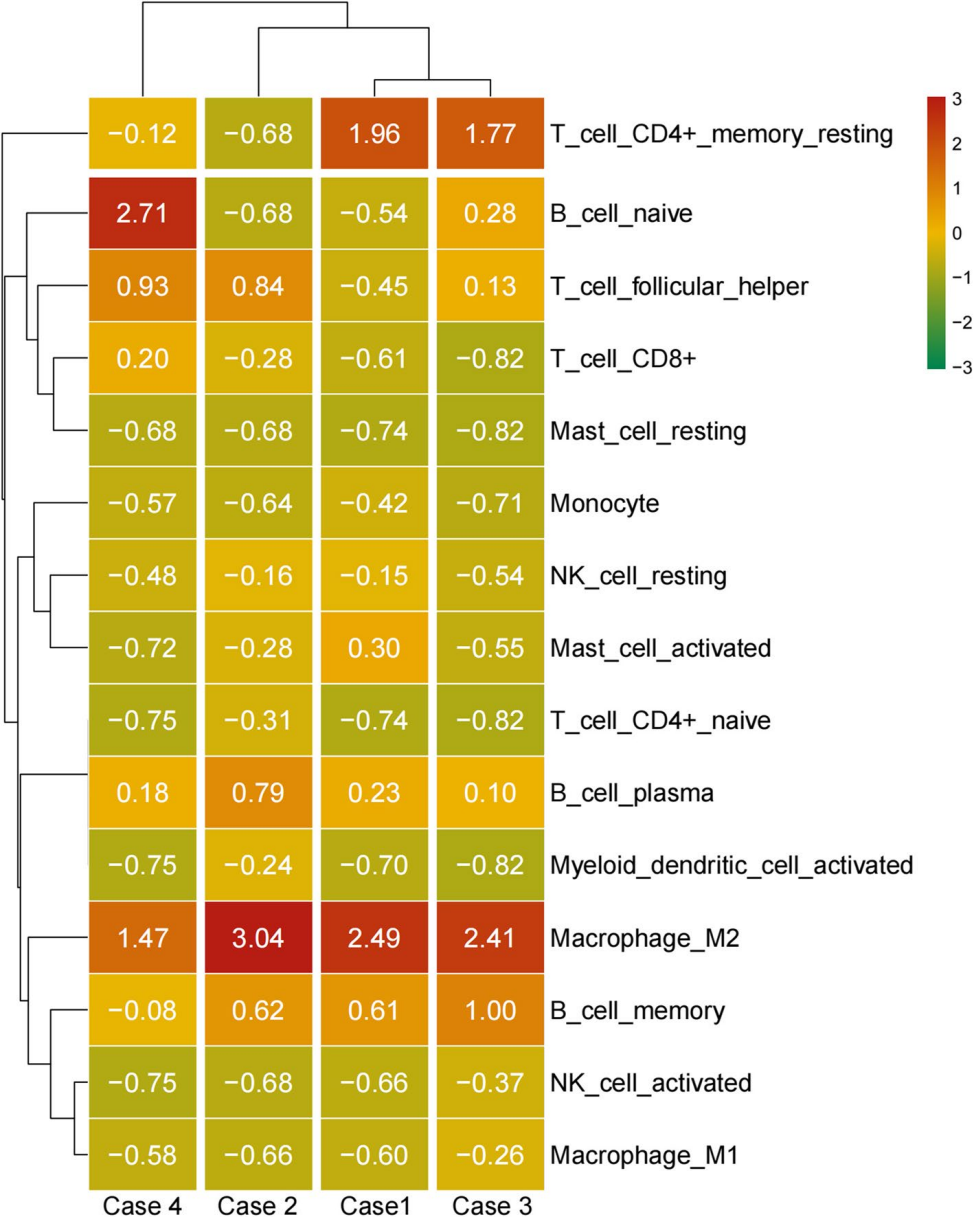
Immune microenvironment status within and across tissue groups. The infiltration percentage of 15 types of immune cells was estimated for each sample with CIBERSORT using gene expression data from RNA sequencing

We systematically reviewed the English language literature published between 1979 and September 2022 in the PubMed and Google Scholar databases and found that only 53 breast MEC cases have been reported (Table 3). All the patients were female with a median age of 56 years old. Among them, 27 (50.9%) were low-grade MEC, 6 (11.3%) were intermediate-grade MEC, 16 (30.2%) were high-grade MEC, and 4 (7.5%) cases were undetermined. Regarding molecular change, we noticed that genetic tests were conducted in 7 reports (8 cases). Four cases, akin to their counterparts arising in the salivary gland, showed *MAML2* rearrangement by FISH, and three cases were confirmed to have *CRTC1-MAML2* fusion by RT-PCR or RNA sequencing. Two cases failed to show *MAML2* rearrangement, while one case showed partial deletion of the 11q21 loci. One report identified a point mutation in *APC*, which was possibly a germline mutation. In our series, three cases were found to have *MAML2* translocation by FISH analysis with *MAML2* break-apart probe (Fig. 3). All four neoplasms were further analyzed by RNA sequencing for fusion partners. Gene fusions were successfully detected in two cases, both harboring the *CRTC1-MAML2* fusion gene (Fig. 4). Case one was negatives for *MAML2* translocation in both FISH detection and RNA Sequencing. Case four was positive by FISH but negative by RNA sequencing (Table 2). Next, we assessed immune cell infiltration levels in these four tumors (Fig. 5). Among 15 immune cells, the infiltration level was heterogeneous among tissue samples. In all these tumors, M2 macrophages had the highest of immune cell infiltration levels. Second, plasma cells were also in the high infiltration group. In contrast, resting mast-cell, monocytes, resting NK cells, CD4 T cells, myeloid-dendritic cells, activated NK cells, M1 Macrophages, and CD8+ T cells all exhibited low infiltration in our series.

**Table 3:**
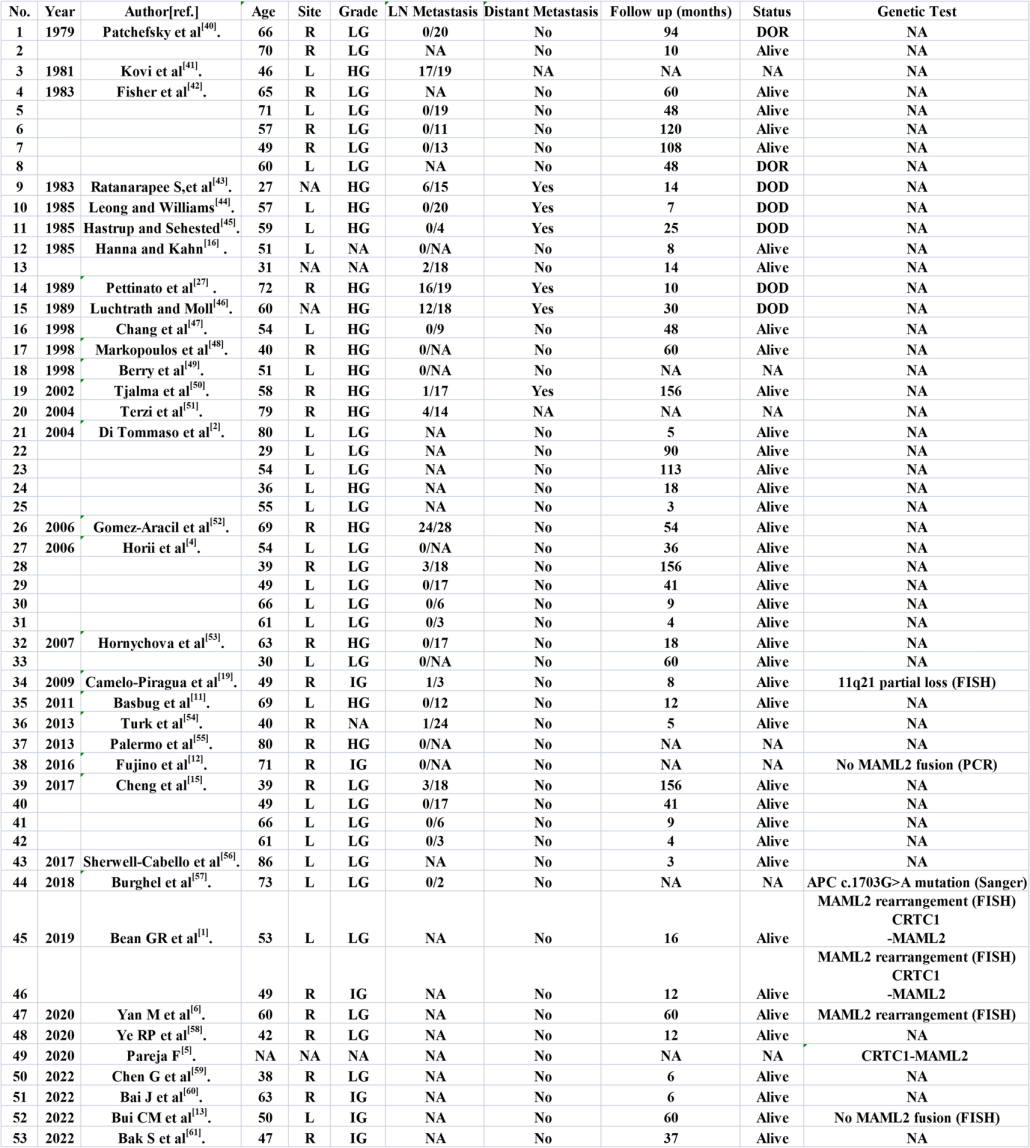
Summary of reported cases of breast mucoepidermoid carcinoma from 1979 to 2022 in English literatures

### Somatic mutations

**Fig. 6:**
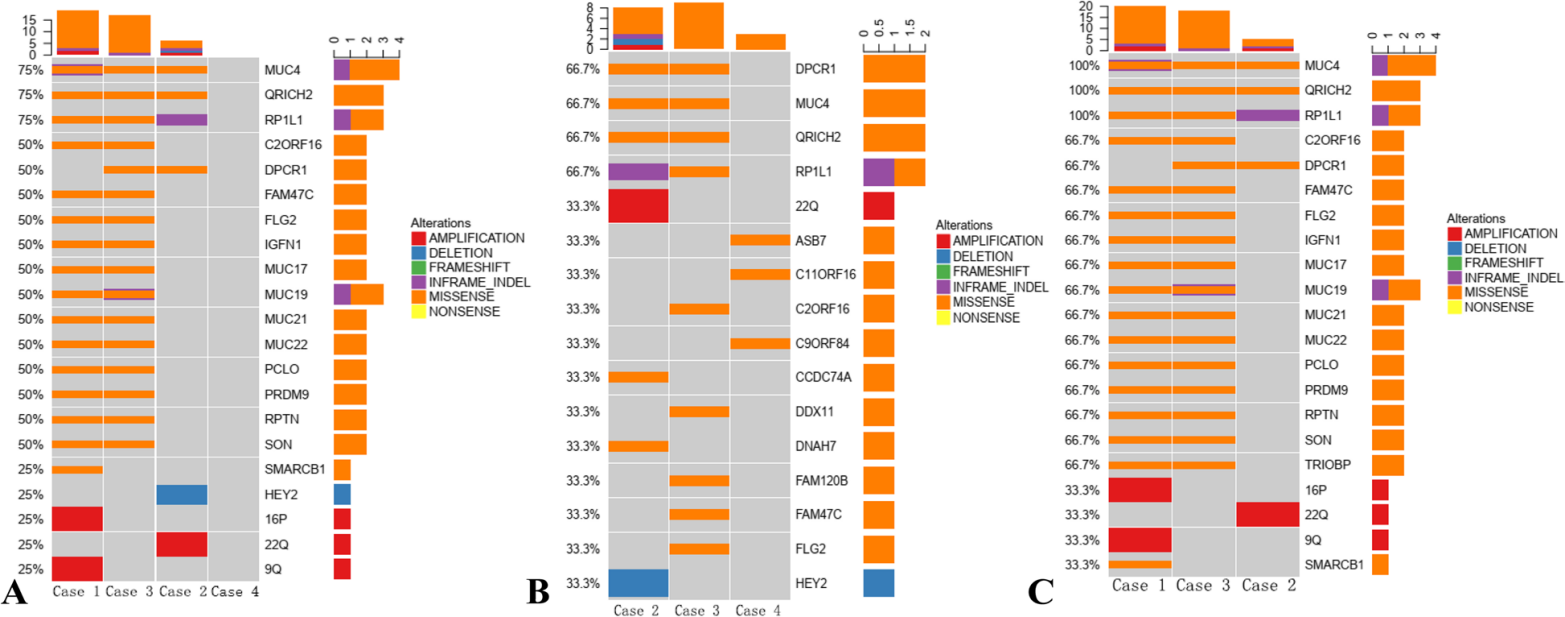
Mutation analysis of breast mucoepidermoid carcinoma patients. Mutation diagram rectangles are colored with respect to the corresponding mutation types. In the case of different types at a single position, colors of the rectangle reflect the two most frequent mutation types. The genes with significant mutations in the samples were arranged according to the mutation frequency. **A** A comutation plot of various types of mutations in breast mucoepidermoid carcinoma patients in our cohort. The cutoff value was 25%. *MUC4*, *QRICH2* and *RP1L1* were found in case one, case two and case three. Case four had a relatively low tumor burden and did not have common mutation genes as the other three cases. **B**, Comparison between case two, case three and case four. The cutoff value was 33.3%. **C**, Comparison between case one, case two and case three. The cutoff value was 33.3%

We identified 245 candidate somatic mutations (241 missense, 4 nonsense) and 10 InDels (7 In_Frame_Dels, 1 In_Frame_Ins, 1 frameshift insertions and 1 frameshift deletion) in 107 genes (supplement Table 1). Mutations per tumor ranged from 8 to 142, with a mean value of 63.75 mutations per tumor. The median TMB of our cases was 2.07 muts/Mb (maximum: 4.53 and minimum: 0.51). A total of 4 CNVs (3 amplifications, 1 deletion) were identified in one *HEY2* gene (loss) and three chromosome amplifications (22q, 9q and 16p). Subsequently, the mutational status of these genes was explored using cBioPortal (www.cbioportal.org) online tool based on TCGA databases. The results showed that there were many genes overlapping with breast invasive carcinoma databases (TCGA, Cell 2015; TCGA, Nature 2012) (supplement Table 2), range from 5 to 63 genes (median:21 genes). *MUC4, RP1L1* and *QRICH2* mutations were identified in at least three tumors and existed in the breast invasive carcinoma databases mentioned above. Among these three genes, *MUC4* was the most frequently mutated gene. There were a total of 29 somatic mutations in *MUC4*, with 1 frameshift alteration and 28 missense mutations. One in-frame insertion and four missense mutations were found in *RP1L1*. All three mutations in *QRICH2* were missense mutations. Case four, however, had a relatively low tumor burden and did not have the same mutation genes as the other three cases (Fig. 6).

## Discussion

MECs are malignant tumors that often occur in the salivary gland. It was first defined by Foote et al. [10] in 1945. Breast and major salivary glands share similar tubule alveolar structures, and both are derived from embryonic ectoderm. Not surprisingly, MECs could also be found in the breast. In this study, we reviewed 1000 breast carcinomas and identified four breast MEC. We also reviewed the English literature published between 1979 and September 2022 in the PubMed and Google Scholar databases and found that only 53 cases have been previously reported. Including the four patients enrolled in our study, all the breast MEC patients were female. Unlike salivary MEC, which was reported to develop secondary to radiation or chemotherapy during childhood, with a median latency period of 8 years [11], the cause of breast MEC is still unknown. One case of mucoepidermoid carcinoma of the breast occurred in a burn scar [12], one was found during radiotherapy and chemotherapy for lymphoma [13], and one was secondary to adenomyoepithelioma [14]. In our study, case two had a history of invasive carcinoma of the contralateral breast and had modified radical mastectomy, followed by chemotherapy and radiation therapy. In our cohort, the median age of the patient was 34.5 years, which is much younger than those in previous studies (median age: 56 years old) [14]. Moreover, it is noteworthy that all the high-grade breast MECs were reported at least 10 years ago. The possible reasons are that the detection technology is improved and people gradually attach importance to routine physical examination. In our series, three patients had no obvious symptoms and nodules were found during the physical examination, while case one was admitted with the first symptom of bloody nipple discharge for more than 1 week.

In previous reports, almost all reported breast MECs were found to be triple-negative breast carcinomas lacking ER and PR expression and *HER2* amplification*.* However, recently, several studies have reported that ER is positive in a subpopulation of breast MECs [4, 14–16]. In the present study, two cases were triple-negative carcinomas. Moreover, both case one and case two showed ER positivity, with case two also displaying HER2 equivocal by immunohistochemical staining but not amplified by FISH. A total of eight patients were confirmed to be ER positive, including our two cases. Five cases were low-grade breast MEC. Two cases were not mentioned about their grades and one showed lymph node metastases but was well without evidence of disease progression [16]. One patient had high-grade breast MEC with lymph node metastases and died of this disease [17]. To date, no solid evidence has shown that ER positivity is related to breast MEC prognosis or grade. Most of them (75%) were Asian people. Several research have demonstrated that racial disparities exist in breast carcinomas, including triple-negative carcinomas [18]. More research is needed to verify whether there are population susceptibility factors in breast MEC.

Bean GR et al. were the first to demonstrate the presence of *CRTC1-MAML2* fusion in breast MEC [1]. The *MAML2* and *CRTC1* genes encode for the Notch/RBPJ mastermind-like 2 and for CREB-regulated transcriptional coactivator 1 proteins, respectively [5]. This translocation might influence the Notch signaling pathway [19]. A recent study showed that salivary gland or lung MEC with this fusion were mostly low-grade tumors and had a better prognosis with a lower risk of local recurrences and metastases [1, 20]. All the cases in our study were low-grade breast MEC. Three of our cases harboring *MAML2* rearrangement were confirmed by FISH, among which two were identified as *CRTC1-MAML2* fusion by RNA sequencing. Histologically, breast MEC is very similar to salivary MEC. However, all tumors located outside the salivary glands shared the same morphological and even immunohistochemical features as MEC of the salivary glands, and MEC of different organs had different prognoses. It seemed that MEC of lung [20], esophagus [21], and thymus [22] had relatively similar prognoses as salivary MEC, and the prognosis was related to the grade and *MAML*2 translocation. However, pancreatic MEC is even more aggressive than ductal adenocarcinoma of the pancreas (PDAC). Almost all pancreatic MEC patients developed lymph node and multiple organ metastases and died within 6 months, except one patient who lived for 45 months [23]. Mucoepidermoid carcinoma of the liver is also regarded as an aggressive tumor with a poor prognosis, as most patients die within 6 months after the initial diagnosis, even with surgical treatment [24]. To date, *MAML2* rearrangement has not been identified in either pancreatic MEC or hepatic MEC. Unlike other MECs, pancreatic MEC and liver MEC retain tissue-specific molecular expression subtypes [23, 24] instead of showing the typical MEC molecular features. In this context, MEC arising in some but not all sites may retain tissue-specific expression patterns, despite otherwise similar morphological features as salivary MEC.

The identification of genetic mutations has become increasingly important since they could serve as treatment targets in precise therapy for cancer and probably improve prognosis. Due to the rarity of the disease, there are very few reports on the molecular characteristics of breast MEC. We first found that low-grade breast MEC had a low mutation burden, which was consistent with that observed in salivary gland MEC*.* Several studies have demonstrated the *TP53* is frequently mutated in intermediate and high-grade salivary gland MEC [25, 26], but is rare in low-grade carcinomas. Hyunseok Kang et al. showed that *POU6F2* was the second most frequently mutated gene and found the same in-frame deletion in three low-grade MECs [26]. However, no *TP53* or *POU6F2* gene mutations were observed in our series.

Somatic alterations in *MUC4, QRICH2* and *PR1L1* were identified in at least three of our breast MECs, which also existed in breast invasive carcinoma databases (TCGA, Cell 2015; TCGA, Nature 2012) and had no relationship with salivary gland MEC [25, 26]. Our series also share many common genes with breast invasive carcinomas, and the median numbers of the same genes was 23.5 and 19, respectively. MUC4 [27] is one of the membrane mucins of the mucin gene (MUC) family, which can modulate cell apoptosis and serve as a modulator of HER2/ ErbB2 signaling. However, in some carcinomas such as salivary gland MEC, overexpression of MUC4 was associated with better prognosis [27]. In the present study, although all four cases were classified as low-grade carcinomas according to Ellis and Auclair’s methods, only case three was MUC4 positive in immunohistochemical staining. To date, there have been few studies on the relationship between MUC4 and breast MEC. Only one case report showed MUC4 expression in two breast MECs [1], which were low-grade and intermediate grade with MUC4 positivity of 20 and 80%, respectively. Therefore, to further verify the relationship between MUC4 and breast MEC prognosis or grades, a study with a larger sample size is needed. *QRICH2* (glutamine rich 2) is located on human chromosome 17 and has been reported to be associated with sperm flagella development and male infertility [28]. Only one case report mentioned that deleterious mutated genes such as *QRICH2* could occur in meningioma [29]. *RP1L1* (retinitis pigmentosa-1-like 1) encodes a component of the photoreceptor axoneme, which is the core structure within the photoreceptor cilium comprised of microtubules and proteins [30]. The associations between *RP1L1* and cancer are basically unknown. Limited studies have reported *RP1L1* mutations in gastric cancer [31]. Additionally, one study showed a relationship between *RP1L1* mutations and dopamine-agonist resistance in prolactinoma [32], and one meta-analysis identified that *PRSS55-RP1L1* was probably associated with the risk of Barrett’s esophagus/esophageal adenocarcinoma in a sex dependent manner [33]. *MUC4, QRICH2* and *PR1L1* mutations were also detected in breast invasive carcinoma databases (TCGA, Cell 2015; TCGA, Nature 2012), but no further research or relationship between these genes and breast carcinomas were published. The biological mechanism of these genes in the pathogenesis of breast MEC needs further investigation.

Infiltration of various types of immune cells into the tumor microenvironment has been shown to play a key role in tumor development. Characterizing the tumor microenvironment and immune landscape of cancer has been a promising step toward discovering new therapeutic biomarkers and guiding precision medicine. Due to its rarity, such efforts have been neglected regarding breast MEC. We profiled the tumor microenvironment in breast MEC using CIBERSORT with respect to 15 immune and stromal cell types. It has been shown that tumors can adjust the microenvironment to survive. Not surprisingly, the documented mediators of direct tumor cell lysis and innate immune cells, such as NK cells and monocytes, were all in the low infiltration group in our series, probably because of consumption in tumor development. CD8+ cytotoxic T cells, which have been clearly established as the ultimate effectors of tumor rejection and could confer long-term protection against cancer recurrence, also had low infiltration [34]. In our series, M2 macrophages and plasma cells belonged to the high infiltration group. M2 macrophages, also known as alternatively activated macrophages, are responsible for tissue remodeling, and angiogenesis usually contributes to tumor growth and metastasis [35]. Unlike M2 cells, M1 macrophages, which usually act as an inflammatory and anticancer factors, had low infiltration in our study. Although the mechanism by which T cells and monocytes regulate tumors has been extensively studied, the role of B cells and their subtypes remains elusive. Depending on phenotypes, antibody isotypes and production, their localization, tumor-infiltrating B and plasma cells had both tumor-promoting and tumor-suppressing characteristics [36]. Hyundeok Kang et al. [37] used RNA sequencing to characterize the tumor microenvironment (TME) and identify immunophenotypic subgroups in salivary gland MEC. In the above study, plasma cells (18/20) were in the low infiltration group among infiltration immune cells, which had no relationship with tumor grade, *MAML2* rearrangement or prognosis. Our breast MEC showed different results from the salivary gland MEC, in which plasma cells were in the high infiltration group. Several studies [38] revealed that plasma cells in TME are implicated in favorable survival rates in breast carcinomas. Furthermore, Yeong et al. [39] revealed that CD38+ plasma cell density was associated with longer disease-free survival independent of clinicopathological parameters in triple-negative carcinomas (TNBCs). This is similar to our results, which are probably related to the microenvironment in mammary glands.

According to our literature review[40–62], most low-grade and intermediate-grade breast MEC had relatively optimistic prognoses, except that one patient with low-grade breast MEC developed high-grade MEC recurrence. Furthermore, among these patients, two died of other reasons, and all of the other patients were alive without disease progression or metastasis (low-grade: median follow-up 41 months including the current four cases, range from 3 to 156 months; intermediate grade: median follow-up 12 months, ranging from 8 to 60 months). Due to the paucity of breast MEC, there is currently no standard treatment. According to the data reported, most patients with low-grade disease had a relatively good overall prognosis. Complete local excision without further adjuvant chemotherapy was probably sufficient to cure the patients [40]. For patients with high-grade malignancy, whole-breast radical surgery and axillary lymph node dissection should be performed [41]. Furthermore, more aggressive protocols, such as chemotherapy, and/or radiotherapy and endocrine therapy, should also be considered, Careful follow-up should be conducted for these patients.

Taken together, although MECs arising in the breast phenotypically resemble their salivary gland counterparts, our findings indicate that at least low-grade breast MECs in Asian people probably resemble invasive breast carcinomas at the genetic level and in the tumor microenvironment. After all, tumors are the product of a very complex and evolutionary process that involves many genes and complicated signaling pathways. Our study could provide some data and ideas for the study of breast MEC. In the future, more cases and especially multicenter cooperation are needed to study the pathogenesis and prognostic factors of breast MEC.

### Supplementary Information

**Additional file 1.**

## Data Availability

No datasets were generated or analysed during the current study.
