# Supplementary material for: Genomics and tumor microenvironment of breast mucoepidermoid carcinoma based on whole-exome and RNA sequencing
Source: Diagn Pathol. 2024 Jan 19;19:15. doi: 10.1186/s13000-024-01439-8 (PMC10797953; doi:10.1186/s13000-024-01439-8)
Supplement: Supplementary file 1 — Additional file 1. [file 13000_2024_1439_MOESM1_ESM.zip › Supplementary Table S2.pdf]

Database_Name	CASE1	CASE2	CASE3	CASE4
Breast Invasive Carcinoma (TCGA, Cell 2015)	TTN	MUC4	MUC4	
Breast Invasive Carcinoma (TCGA, Cell 2015)	MUC4	DNAH7	MUC17	PSME4
Breast Invasive Carcinoma (TCGA, Cell 2015)	DMD	QRICH2	PCLO	PLEKHM2
Breast Invasive Carcinoma (TCGA, Cell 2015)	MUC17	RP1L1	FLG2	ASB7
Breast Invasive Carcinoma (TCGA, Cell 2015)	PCLO	PABPC3	ZNF208	RFX2
Breast Invasive Carcinoma (TCGA, Cell 2015)	PAPPA2	CCDC74A	FAM47C	ZNF668
Breast Invasive Carcinoma (TCGA, Cell 2015)	ZAN	ZNF479	DDX11	
Breast Invasive Carcinoma (TCGA, Cell 2015)	FLG2	TOX4	QRICH2	
Breast Invasive Carcinoma (TCGA, Cell 2015)	MUC2	PRODH	GOLGA6L6	
Breast Invasive Carcinoma (TCGA, Cell 2015)	TCHH	WBP2NL	RP1L1	
Breast Invasive Carcinoma (TCGA, Cell 2015)	ZNF208	UGT1A9	RPTN	
Breast Invasive Carcinoma (TCGA, Cell 2015)	RBMXL3		IGFN1	
Breast Invasive Carcinoma (TCGA, Cell 2015)	FHOD3		ZNF91	
Breast Invasive Carcinoma (TCGA, Cell 2015)	FAM47C		MYO15A	
Breast Invasive Carcinoma (TCGA, Cell 2015)	FAM186A		TUBGCP6	
Breast Invasive Carcinoma (TCGA, Cell 2015)	QRICH2		ZNF99	
Breast Invasive Carcinoma (TCGA, Cell 2015)	AKAP11		SON	
Breast Invasive Carcinoma (TCGA, Cell 2015)	RP1L1		PRB2	
Breast Invasive Carcinoma (TCGA, Cell 2015)	RPTN		ZNF681	
Breast Invasive Carcinoma (TCGA, Cell 2015)	ZNF43		MUC19	
Breast Invasive Carcinoma (TCGA, Cell 2015)	DSPP		MDC1	
Breast Invasive Carcinoma (TCGA, Cell 2015)	OR4A15		PRDM9	
Breast Invasive Carcinoma (TCGA, Cell 2015)	IGFN1		ZNF90	
Breast Invasive Carcinoma (TCGA, Cell 2015)	MAGEC1		NEFH	
Breast Invasive Carcinoma (TCGA, Cell 2015)	ZNF91		TRIOBP	
Breast Invasive Carcinoma (TCGA, Cell 2015)	ZNF729		FAM120B	
Breast Invasive Carcinoma (TCGA, Cell 2015)	PRG4		ZNF726	
Breast Invasive Carcinoma (TCGA, Cell 2015)	ZNF99		TOP3B	
Breast Invasive Carcinoma (TCGA, Cell 2015)	SON		KRT1	
Breast Invasive Carcinoma (TCGA, Cell 2015)	ZNF479		ZNF737	
Breast Invasive Carcinoma (TCGA, Cell 2015)	POTEE		GOLGA6L1	
Breast Invasive Carcinoma (TCGA, Cell 2015)	ZNF681		MCM5	
Breast Invasive Carcinoma (TCGA, Cell 2015)	MUC19		MUC21	
Breast Invasive Carcinoma (TCGA, Cell 2015)	PRDM9		RIMBP3B	
Breast Invasive Carcinoma (TCGA, Cell 2015)	ZNF90		MADCAM1	
Breast Invasive Carcinoma (TCGA, Cell 2015)	PRKCB		ZNF430	
Breast Invasive Carcinoma (TCGA, Cell 2015)	TRIOBP			
Breast Invasive Carcinoma (TCGA, Cell 2015)	TXNDC2			
Breast Invasive Carcinoma (TCGA, Cell 2015)	ZNF726			
Breast Invasive Carcinoma (TCGA, Cell 2015)	ZNF534			
Breast Invasive Carcinoma (TCGA, Cell 2015)	PKP1			
Breast Invasive Carcinoma (TCGA, Cell 2015)	ZNF737			
Breast Invasive Carcinoma (TCGA, Cell 2015)	ZNF714			
Breast Invasive Carcinoma (TCGA, Cell 2015)	ZBTB22			
Breast Invasive Carcinoma (TCGA, Cell 2015)	CCDC8			
Breast Invasive Carcinoma (TCGA, Cell 2015)	ADAR			
Breast Invasive Carcinoma (TCGA, Cell 2015)	SMARCB1			
Breast Invasive Carcinoma (TCGA, Cell 2015)	PRB3			
Breast Invasive Carcinoma (TCGA, Cell 2015)	PTGFRN			
Breast Invasive Carcinoma (TCGA, Cell 2015)	FAHD2B			
Breast Invasive Carcinoma (TCGA, Cell 2015)	ZNF100			
Breast Invasive Carcinoma (TCGA, Cell 2015)	ZNF492			
Breast Invasive Carcinoma (TCGA, Cell 2015)	SELPLG			

Breast Invasive Carcinoma (TCGA, Cell 2015)	SBSN			
Breast Invasive Carcinoma (TCGA, Cell 2015)	GOLGA6L10			
Breast Invasive Carcinoma (TCGA, Cell 2015)	SNRNP27			
Breast Invasive Carcinoma (TCGA, Cell 2015)	CCDC157			
Breast Invasive Carcinoma (TCGA, Cell 2015)	MUC21			
Breast Invasive Carcinoma (TCGA, Cell 2015)	ZNF430			
Breast Invasive Carcinoma (TCGA, Cell 2015)	SRRM5			
Breast Invasive Carcinoma (TCGA, Cell 2015)	DNAAF1			
Breast Invasive Carcinoma (TCGA, Cell 2015)	ZNF765			
Breast Invasive Carcinoma (TCGA, Cell 2015)	ZNF92			
Breast Invasive Carcinoma (TCGA, Nature 2012)	SRRM5	QRICH2	TUBGCP6	PLEKHM2
Breast Invasive Carcinoma (TCGA, Nature 2012)	QRICH2	TOX4	QRICH2	PSME4
Breast Invasive Carcinoma (TCGA, Nature 2012)	TRIOBP	PABPC3	TRIOBP	ZNF668
Breast Invasive Carcinoma (TCGA, Nature 2012)	TTN	RP1L1	IGFN1	ASB7
Breast Invasive Carcinoma (TCGA, Nature 2012)	IGFN1	ZNF479	KRT1	RFX2
Breast Invasive Carcinoma (TCGA, Nature 2012)	DNAAF1	DNAH7	FLG2	
Breast Invasive Carcinoma (TCGA, Nature 2012)	AKAP11	MUC4	MUC17	
Breast Invasive Carcinoma (TCGA, Nature 2012)	MAGEC1		TOP3B	
Breast Invasive Carcinoma (TCGA, Nature 2012)	FLG2		FAM47C	
Breast Invasive Carcinoma (TCGA, Nature 2012)	TXNDC2		ZNF91	
Breast Invasive Carcinoma (TCGA, Nature 2012)	ZAN		ZNF90	
Breast Invasive Carcinoma (TCGA, Nature 2012)	ZNF100		ZNF99	
Breast Invasive Carcinoma (TCGA, Nature 2012)	ZNF43		FAM120B	
Breast Invasive Carcinoma (TCGA, Nature 2012)	MUC17		MUC21	
Breast Invasive Carcinoma (TCGA, Nature 2012)	ZNF765		ZNF208	
Breast Invasive Carcinoma (TCGA, Nature 2012)	FAM47C		DDX11	
Breast Invasive Carcinoma (TCGA, Nature 2012)	ZNF91		ZNF728	
Breast Invasive Carcinoma (TCGA, Nature 2012)	ZNF90		ZNF850	
Breast Invasive Carcinoma (TCGA, Nature 2012)	PRG4		SON	
Breast Invasive Carcinoma (TCGA, Nature 2012)	ZNF99		RP1L1	
Breast Invasive Carcinoma (TCGA, Nature 2012)	MUC21		PRB2	
Breast Invasive Carcinoma (TCGA, Nature 2012)	SELPLG		MYO15A	
Breast Invasive Carcinoma (TCGA, Nature 2012)	ZNF208		PCLO	
Breast Invasive Carcinoma (TCGA, Nature 2012)	ZNF728		ZNF726	
Breast Invasive Carcinoma (TCGA, Nature 2012)	ZNF850		ZNF681	
Breast Invasive Carcinoma (TCGA, Nature 2012)	FAM186A		GOLGA6L1	
Breast Invasive Carcinoma (TCGA, Nature 2012)	ZNF534		MUC4	
Breast Invasive Carcinoma (TCGA, Nature 2012)	DMD		MADCAM1	
Breast Invasive Carcinoma (TCGA, Nature 2012)	PAPPA2		MDC1	
Breast Invasive Carcinoma (TCGA, Nature 2012)	SON		ZNF430	
Breast Invasive Carcinoma (TCGA, Nature 2012)	PKP1		RPTN	
Breast Invasive Carcinoma (TCGA, Nature 2012)	RP1L1			
Breast Invasive Carcinoma (TCGA, Nature 2012)	DSPP			
Breast Invasive Carcinoma (TCGA, Nature 2012)	PCLO			
Breast Invasive Carcinoma (TCGA, Nature 2012)	ZNF729			
Breast Invasive Carcinoma (TCGA, Nature 2012)	PRB3			
Breast Invasive Carcinoma (TCGA, Nature 2012)	ZNF479			
Breast Invasive Carcinoma (TCGA, Nature 2012)	ZNF726			
Breast Invasive Carcinoma (TCGA, Nature 2012)	PRKCB			
Breast Invasive Carcinoma (TCGA, Nature 2012)	ZNF681			
Breast Invasive Carcinoma (TCGA, Nature 2012)	SBSN			
Breast Invasive Carcinoma (TCGA, Nature 2012)	ZBTB22			
Breast Invasive Carcinoma (TCGA, Nature 2012)	PTGFRN			
Breast Invasive Carcinoma (TCGA, Nature 2012)	ZNF492			

Breast Invasive Carcinoma (TCGA, Nature 2012)	RBMXL3
Breast Invasive Carcinoma (TCGA, Nature 2012)	MUC2
Breast Invasive Carcinoma (TCGA, Nature 2012)	MUC4
Breast Invasive Carcinoma (TCGA, Nature 2012)	OR4A15
Breast Invasive Carcinoma (TCGA, Nature 2012)	CCDC8
Breast Invasive Carcinoma (TCGA, Nature 2012)	FHOD3
Breast Invasive Carcinoma (TCGA, Nature 2012)	POTEE
Breast Invasive Carcinoma (TCGA, Nature 2012)	TCHH
Breast Invasive Carcinoma (TCGA, Nature 2012)	ZNF430
Breast Invasive Carcinoma (TCGA, Nature 2012)	RPTN
Breast Invasive Carcinoma (TCGA, Nature 2012)	ADAR
