# Supplementary material for: Genomics and tumor microenvironment of breast mucoepidermoid carcinoma based on whole-exome and RNA sequencing
Source: Diagn Pathol. 2024 Jan 19;19:15. doi: 10.1186/s13000-024-01439-8 (PMC10797953; doi:10.1186/s13000-024-01439-8)
Supplement: Supplementary file 1 — Additional file 1. [file 13000_2024_1439_MOESM1_ESM.zip › Supplementary Table S1.pdf]

Sample_ID	Gene_Symbol	Chromosome	Variant_Classification
1	MUC4	3	Missense_Mutation
1	MUC4	3	Missense_Mutation
1	RP1L1	8	Missense_Mutation
1	PTGFRN	1	Missense_Mutation
1	TCHH	1	Missense_Mutation
1	CCDC8	19	Missense_Mutation
1	TXNDC2	18	Missense_Mutation
1	DSPP	4	Missense_Mutation
1	MUC2	11	Missense_Mutation
1	MUC17	7	Missense_Mutation
1	MUC4	3	Missense_Mutation
1	RP1L1	8	Missense_Mutation
1	ZNF681	19	Missense_Mutation
1	FAM186A	12	Missense_Mutation
1	KRTAP10-6	21	Missense_Mutation
1	POTEE	2	Missense_Mutation
1	AKAP11	13	Nonsense_Mutation
1	FHOD3	18	Missense_Mutation
1	MUC21	6	Missense_Mutation
1	PRDM9	5	Missense_Mutation
1	ZNF92	7	Missense_Mutation
1	ZNF714	19	Missense_Mutation
1	KIAA1239	4	Missense_Mutation
1	PRB3	12	Missense_Mutation
1	SMARCB1	22	Missense_Mutation
1	LOC101928841	13	Missense_Mutation
1	DSPP	4	Missense_Mutation
1	MUC4	3	Missense_Mutation
1	RBMXL3	X	Missense_Mutation
1	TXNDC2	18	Missense_Mutation
1	C2orf16	2	Missense_Mutation
1	FLG2	1	Missense_Mutation
1	MUC4	3	In_Frame_Del
1	MUC4	3	Missense_Mutation
1	ZNF850	19	Missense_Mutation
1	KRTAP12-2	21	Missense_Mutation
1	MUC4	3	Missense_Mutation
1	GOLGA6L10	15	Missense_Mutation
1	ZNF43	19	Missense_Mutation
1	RPTN	1	Missense_Mutation
1	SNRNP27	2	Missense_Mutation
1	ZNF728	19	Missense_Mutation
1	MUC17	7	Missense_Mutation
1	MUC22	6	Missense_Mutation
1	IGFN1	1	Missense_Mutation
1	SRRM5	19	Missense_Mutation
1	QRICH2	17	Missense_Mutation
1	ZNF208	19	Missense_Mutation
1	MUC4	3	Missense_Mutation
1	ZNF714	19	Missense_Mutation
1	MUC22	6	Missense_Mutation
1	ZNF765	19	Missense_Mutation
1	MUC19	12	Missense_Mutation

1	DNAAF1	16	Missense_Mutation
1	MUC4	3	Missense_Mutation
1	MUC19	12	Missense_Mutation
1	ZNF534	19	Missense_Mutation
1	SLC9B1	4	Nonsense_Mutation
1	MUC19	12	Missense_Mutation
1	FAM186A	12	Missense_Mutation
1	SON	21	Missense_Mutation
1	TXNDC2	18	Missense_Mutation
1	ZNF90	19	Missense_Mutation
1	ZNF99	19	Missense_Mutation
1	ZNF729	19	Missense_Mutation
1	PRG4	1	Missense_Mutation
1	ZNF479	7	Missense_Mutation
1	ZNF208	19	Missense_Mutation
1	DSPP	4	Missense_Mutation
1	FLG2	1	Missense_Mutation
1	ADAR	1	Missense_Mutation
1	MUC4	3	Missense_Mutation
1	PKP1	1	Missense_Mutation
1	MUC4	3	Missense_Mutation
1	FAM186A	12	Missense_Mutation
1	ZAN	7	Missense_Mutation
1	MUC4	3	Missense_Mutation
1	ZNF208	19	Missense_Mutation
1	TRIOBP	22	Missense_Mutation
1	PAPPA2	1	Missense_Mutation
1	ZBTB22	6	Missense_Mutation
1	MUC19	12	Missense_Mutation
1	ZNF492	19	Missense_Mutation
1	IGFN1	1	Missense_Mutation
1	MUC19	12	Missense_Mutation
1	PRKCB	16	Missense_Mutation
1	DSPP	4	Missense_Mutation
1	LOC101928841	13	Missense_Mutation
1	PCLO	7	Missense_Mutation
1	MUC4	3	Missense_Mutation
1	GOLGA6L10	15	Missense_Mutation
1	ZNF729	19	Missense_Mutation
1	PRG4	1	Missense_Mutation
1	LOC101928841	13	Missense_Mutation
1	LOC101928841	13	Missense_Mutation
1	MUC4	3	Missense_Mutation
1	OR4A15	11	Missense_Mutation
1	MAGEC1	X	Missense_Mutation
1	MUC22	6	Missense_Mutation
1	MUC21	6	Missense_Mutation
1	MUC19	12	Missense_Mutation
1	MUC4	3	Missense_Mutation
1	ACRC	X	Missense_Mutation
1	ZNF91	19	Missense_Mutation
1	MUC21	6	Missense_Mutation
1	PRG4	1	Missense_Mutation
1	MUC4	3	Missense_Mutation

1	FAM186A	12	Missense_Mutation
1	ZNF726	19	Missense_Mutation
1	RPTN	1	Missense_Mutation
1	ZNF737	19	Missense_Mutation
1	RPTN	1	Missense_Mutation
1	TTN	2	Missense_Mutation
1	MUC4	3	Missense_Mutation
1	SELPLG	12	Missense_Mutation
1	FAM186A	12	Missense_Mutation
1	MUC4	3	Missense_Mutation
1	DSPP	4	Missense_Mutation
1	FAHD2B	2	Missense_Mutation
1	DMD	X	Nonsense_Mutation
1	SRRM5	19	Missense_Mutation
1	ZNF430	19	Missense_Mutation
1	ZNF43	19	Missense_Mutation
1	ZNF100	19	Missense_Mutation
1	MUC4	3	Missense_Mutation
1	FAM47C	X	Missense_Mutation
1	MUC19	12	Missense_Mutation
1	MUC4	3	Missense_Mutation
1	MUC17	7	Missense_Mutation
1	ZNF681	19	Missense_Mutation
1	MUC22	6	Missense_Mutation
1	MUC4	3	Missense_Mutation
1	RPTN	1	Missense_Mutation
1	MUC2	11	Missense_Mutation
1	GOLGA6L10	15	Missense_Mutation
1	SBSN	19	Missense_Mutation
1	MUC4	3	Missense_Mutation
1	FAM47C	X	Missense_Mutation
1	MUC19	12	Missense_Mutation
1	RP1L1	8	Missense_Mutation
1	CCDC157	22	Missense_Mutation
1	PRB3	12	Missense_Mutation
2	CCDC74A	2	Missense_Mutation
2	DNAH7	2	Missense_Mutation
2	DPCR1	6	Missense_Mutation
2	LOC101928841	13	In_Frame_Del
2	LOC101928841	13	Missense_Mutation
2	MUC4	3	Missense_Mutation
2	MUC4	3	Missense_Mutation
2	PABPC3	13	Nonsense_Mutation
2	PRODH	22	Missense_Mutation
2	QRICH2	17	Missense_Mutation
2	RP1L1	8	In_Frame_Ins
2	TOX4	14	Frame_Shift_Del
2	UGT1A9	2	Missense_Mutation
2	WBP2NL	22	In_Frame_Del
2	ZNF479	7	Missense_Mutation
3	MUC22	6	Missense_Mutation
3	FLG2	1	Missense_Mutation
3	PRB2	12	Missense_Mutation
3	MYO15A	17	Missense_Mutation

3	FLG2	1	Missense_Mutation
3	DPCR1	6	Missense_Mutation
3	FAM47C	X	Missense_Mutation
3	ZNF726	19	Missense_Mutation
3	FLG2	1	Missense_Mutation
3	MUC17	7	Missense_Mutation
3	ZNF681	19	Missense_Mutation
3	LOC101928841	13	Missense_Mutation
3	MUC19	12	Missense_Mutation
3	DPCR1	6	Missense_Mutation
3	NEFH	22	In_Frame_Del
3	ZNF737	19	Missense_Mutation
3	ZNF850	19	Missense_Mutation
3	ZNF208	19	Missense_Mutation
3	ZNF91	19	Missense_Mutation
3	ZNF728	19	Missense_Mutation
3	MUC22	6	Missense_Mutation
3	MUC19	12	Missense_Mutation
3	MUC4	3	Missense_Mutation
3	C2orf16	2	Missense_Mutation
3	MUC19	12	Missense_Mutation
3	ZNF208	19	Missense_Mutation
3	PCLO	7	Missense_Mutation
3	QRICH2	17	Missense_Mutation
3	PRDM9	5	Missense_Mutation
3	ZNF726	19	Missense_Mutation
3	MADCAM1	19	Missense_Mutation
3	MUC19	12	Missense_Mutation
3	MCM5	22	Missense_Mutation
3	SON	21	Missense_Mutation
3	MUC19	12	Missense_Mutation
3	MUC19	12	Missense_Mutation
3	FAM47C	X	Missense_Mutation
3	PCLO	7	Missense_Mutation
3	NPIPB5	16	Missense_Mutation
3	MUC19	12	Missense_Mutation
3	HGC6.3	6	Missense_Mutation
3	MUC4	3	Missense_Mutation
3	ZNF208	19	Missense_Mutation
3	TUBGCP6	22	Missense_Mutation
3	ZNF430	19	Missense_Mutation
3	ZNF728	19	Missense_Mutation
3	IGFN1	1	Missense_Mutation
3	FAM120B	6	Missense_Mutation
3	ZNF737	19	Missense_Mutation
3	MUC19	12	Missense_Mutation
3	RPTN	1	Missense_Mutation
3	DPCR1	6	Missense_Mutation
3	TRIOBP	22	Missense_Mutation
3	MDC1	6	Missense_Mutation
3	KRT1	12	Missense_Mutation
3	MUC4	3	Missense_Mutation
3	GOLGA6L1	15	Missense_Mutation
3	MUC19	12	In_Frame_Del

3	RP1L1	8	Missense_Mutation
3	LOC101928841	13	Missense_Mutation
3	ZFP112	19	Missense_Mutation
3	TOP3B	22	Missense_Mutation
3	SDF2L1	22	Missense_Mutation
3	MUC22	6	Missense_Mutation
3	FLG2	1	Missense_Mutation
3	RPTN	1	Missense_Mutation
3	ZNF90	19	Missense_Mutation
3	ZNF99	19	Missense_Mutation
3	MUC22	6	Missense_Mutation
3	MUC19	12	Missense_Mutation
3	RP1L1	8	Missense_Mutation
3	MUC19	12	Missense_Mutation
3	MUC4	3	Missense_Mutation
3	DDX11	12	Missense_Mutation
3	ZNF99	19	Missense_Mutation
3	MUC4	3	Missense_Mutation
3	ZNF90	19	Missense_Mutation
3	MUC4	3	Missense_Mutation
3	MUC21	6	Missense_Mutation
3	RIMBP3B	22	Missense_Mutation
3	GOLGA6L6	15	Missense_Mutation
3	FLG2	1	Missense_Mutation
3	MUC17	7	Missense_Mutation
3	ZNF728	19	Missense_Mutation
3	RPTN	1	Missense_Mutation
3	MUC17	7	Missense_Mutation
3	ZNF99	19	Missense_Mutation
3	DPCR1	6	Missense_Mutation
3	NEFH	22	In_Frame_Del
3	ZNF99	19	Missense_Mutation
4	RFX2	19	Missense_Mutation
4	C11orf16	11	Missense_Mutation
4	C9orf84	9	Missense_Mutation
4	ZNF668	16	In_Frame_Del
4	ASB7	15	Missense_Mutation
4	PSME4	2	Missense_Mutation
4	PLEKHM2	1	Missense_Mutation
4	NUPL2	7	Frame_Shift_Ins

Transcript_ID
NM_018406.6
NM_018406.6
NM_178857.5
NM_020440.2
NM_007113.3
NM_032040.4
NM_001098529.1
NM_014208.3
NM_002457.2
NM_001040105.1
NM_018406.6
NM_178857.5
NM_138286.2
NM_001145475.1
NM_198688.2
NM_001083538.1
NM_016248.3
NM_001281740.2
NM_001010909.2
NM_020227.2
NM_152626.2
NM_182515.3
NM_001144990.1
NM_006249.4
NM_003073.3
NM_001304433.1
NM_014208.3
NM_018406.6
NM_001145346.1
NM_001098529.1
NM_032266.3
NM_001014342.2
NM_018406.6
NM_018406.6
NM_001267779.1
NM_181684.2
NM_018406.6
NM_001164465.3
NM_003423.3
NM_001122965.1
NM_006857.2
NM_001267716.1
NM_001040105.1
NM_001198815.1
NM_001164586.1
NM_001145641.1
NM_032134.1
NM_007153.3
NM_018406.6
NM_182515.3
NM_001198815.1
NM_001040185.1
NM_173600.2

NM_178452.4
NM_018406.6
NM_173600.2
NM_001143939.1
NM_139173.3
NM_173600.2
NM_001145475.1
NM_138927.2
NM_001098529.1
NM_007138.1
NM_001080409.2
NM_001242680.1
NM_005807.3
NM_033273.2
NM_007153.3
NM_014208.3
NM_001014342.2
NM_015840.3
NM_018406.6
NM_000299.3
NM_018406.6
NM_001145475.1
NM_003386.1
NM_018406.6
NM_007153.3
NM_001039141.2
NM_020318.2
NM_001145338.1
NM_173600.2
NM_020855.2
NM_001164586.1
NM_173600.2
NM_002738.6
NM_014208.3
NM_001304433.1
NM_033026.5
NM_018406.6
NM_001164465.3
NM_001242680.1
NM_005807.3
NM_001304433.1
NM_001304433.1
NM_018406.6
NM_001005275.1
NM_005462.4
NM_001198815.1
NM_001010909.2
NM_173600.2
NM_018406.6
NM_052957.4
NM_003430.2
NM_001010909.2
NM_005807.3
NM_018406.6

NM_001145475.1
NM_001244038.1
NM_001122965.1
NM_001159293.1
NM_001122965.1
NM_001256850.1
NM_018406.6
NM_003006.4
NM_001145475.1
NM_018406.6
NM_014208.3
NM_199336.1
NM_004011.3
NM_001145641.1
NM_001172671.1
NM_003423.3
NM_173531.3
NM_018406.6
NM_001013736.2
NM_173600.2
NM_018406.6
NM_001040105.1
NM_138286.2
NM_001198815.1
NM_018406.6
NM_001122965.1
NM_002457.2
NM_001164465.3
NM_001166034.1
NM_018406.6
NM_001013736.2
NM_173600.2
NM_178857.5
NM_001017437.2
NM_006249.4
NM_001258304.1
NM_018897.2
NM_080870.3
NM_001304433.1
NM_001304433.1
NM_018406.6
NM_018406.6
NM_030979.2
NM_016335.4
NM_032134.1
NM_178857.5
NM_014828.3
NM_021027.2
NM_152613.2
NM_033273.2
NM_001198815.1
NM_001014342.2
NM_006248.3
NM_016239.3

NM_001014342.2
NM_080870.3
NM_001013736.2
NM_001244038.1
NM_001014342.2
NM_001040105.1
NM_138286.2
NM_001304433.1
NM_173600.2
NM_080870.3
NM_021076.3
NM_001159293.1
NM_001267779.1
NM_007153.3
NM_003430.2
NM_001267716.1
NM_001198815.1
NM_173600.2
NM_018406.6
NM_032266.3
NM_173600.2
NM_007153.3
NM_033026.5
NM_032134.1
NM_020227.2
NM_001244038.1
NM_130760.2
NM_173600.2
NM_006739.3
NM_138927.2
NM_173600.2
NM_173600.2
NM_001013736.2
NM_033026.5
NM_001135865.1
NM_173600.2
NM_001129895.2
NM_018406.6
NM_007153.3
NM_020461.3
NM_001172671.1
NM_001267716.1
NM_001164586.1
NM_001286379.1
NM_001159293.1
NM_173600.2
NM_001122965.1
NM_080870.3
NM_001039141.2
NM_014641.2
NM_006121.3
NM_018406.6
NM_001001413.3
NM_173600.2

NM_178857.5
NM_001304433.1
NM_001083335.1
NM_001282112.1
NM_022044.2
NM_001198815.1
NM_001014342.2
NM_001122965.1
NM_007138.1
NM_001080409.2
NM_001198815.1
NM_173600.2
NM_178857.5
NM_173600.2
NM_018406.6
NM_001257144.1
NM_001080409.2
NM_018406.6
NM_007138.1
NM_018406.6
NM_001010909.2
NM_001128635.1
NM_001145004.1
NM_001014342.2
NM_001040105.1
NM_001267716.1
NM_001122965.1
NM_001040105.1
NM_001080409.2
NM_080870.3
NM_021076.3
NM_001080409.2
NM_000635.4
NM_020643.3
NM_173521.5
NM_024706.5
NM_024708.3
NM_014614.3
NM_015164.4
NM_007342.3

HGVSc
c.12236G>C
c.5518T>C
c.6490A>G
c.2275C>T
c.3072C>G
c.1132C>G
c.1135A>G
c.2854A>G
c.5089T>C
c.8536A>G
c.10220T>C
c.6258T>G
c.1475T>A
c.3688A>C
c.1042A>G
c.196C>T
c.4990C>T
c.4703G>A
c.1148T>C
c.1696C>G
c.1166G>T
c.1304G>A
c.1762G>A
c.284G>C
c.1129C>T
c.4010G>A
c.2657A>G
c.12238A>C
c.1850G>A
c.844G>A
c.5319A>C
c.1864A>G
c.3050_3097del
c.5415G>C
c.1297C>A
c.166C>G
c.11728A>C
c.856C>T
c.1778T>C
c.1606A>G
c.19C>T
c.984G>T
c.8868G>C
c.3016T>A
c.6394A>G
c.1640A>G
c.1859A>C
c.3553G>A
c.5533A>G
c.1268T>C
c.3230T>C
c.1108T>C
c.11354T>C

c.1306G>C
c.5566T>C
c.11267T>C
c.1717A>C
c.1318A>T
c.11302T>C
c.3481A>C
c.2398T>A
c.709A>G
c.971T>G
c.2398A>C
c.1514G>A
c.2357T>C
c.1313A>C
c.3839T>C
c.2795A>G
c.2246G>T
c.805T>G
c.9903G>C
c.2138C>T
c.8975T>C
c.4295A>G
c.1930T>A
c.5437A>G
c.3213A>C
c.2082G>C
c.2569A>G
c.461C>T
c.11356G>A
c.118G>A
c.5152G>A
c.11653T>A
c.472C>T
c.2775A>C
c.4166G>A
c.1618T>C
c.11306T>C
c.667T>C
c.1943C>T
c.1417G>A
c.3974G>A
c.3938G>A
c.5551T>A
c.32T>A
c.1080T>A
c.3584T>C
c.833T>C
c.13955A>C
c.5614T>C
c.563T>C
c.1025G>T
c.304A>G
c.1249A>C
c.11113T>G

c.3455A>C
c.1000C>A
c.1254G>A
c.1058T>C
c.1469T>C
c.3988C>T
c.11341G>A
c.563G>C
c.4007A>G
c.12401T>C
c.3103G>A
c.682G>A
c.4857G>A
c.1525T>C
c.1410A>C
c.1777T>C
c.1347G>A
c.6904G>A
c.1391T>C
c.11471A>G
c.5777T>C
c.1549T>C
c.1513T>C
c.3254T>C
c.11872A>C
c.1481A>G
c.5828C>T
c.792_793inv
c.722A>G
c.10670A>G
c.1747T>C
c.11384T>C
c.6452A>C
c.2092T>C
c.202T>C
c.263G>A
c.2239G>A
c.2237G>C
c.4127_4165delinsGTT
c.4276A>G
c.10097T>C
c.4605G>C
c.1033G>T
c.1465G>A
c.1900G>A
c.6489_6490delinsGGATGCCCAGCCAGAGTCAGACGGTG
c.108del
c.505G>A
c.559_579del
c.1313A>C
c.3617T>C
c.1940C>T
c.703G>T
c.6917G>A

c.6171A>C
c.2995A>G
c.1622G>C
c.1375A>G
c.2837T>C
c.1412A>C
c.1513T>C
c.3892A>G
c.14204T>C
c.1421T>C
c.1973_1978del
c.1058T>C
c.1141G>C
c.3121C>T
c.1081A>C
c.1210A>G
c.3778T>A
c.14044G>A
c.5437A>G
c.5415A>C
c.16039A>G
c.3213A>C
c.1618T>C
c.1900G>A
c.2033T>A
c.1492G>A
c.713C>A
c.13879T>G
c.1312G>A
c.2338T>A
c.15692T>C
c.12893A>G
c.1391T>C
c.1489G>A
c.1846T>G
c.14152A>G
c.101T>C
c.3229A>G
c.1246G>A
c.3532A>G
c.923G>T
c.984G>T
c.6394A>G
c.1333C>T
c.1148A>T
c.17189A>G
c.1426A>G
c.2567T>C
c.1543A>G
c.4751G>C
c.340A>T
c.6506C>T
c.992G>A
c.11354_11384delinsC

c.6130A>G
c.3974G>A
c.1778A>G
c.2299T>C
c.335G>A
c.3392T>C
c.2093T>C
c.1481A>G
c.1400A>T
c.2248G>A
c.3584T>C
c.11539A>G
c.6452A>C
c.11537T>C
c.7528A>G
c.500G>C
c.1852A>G
c.12670C>T
c.1474T>C
c.4766A>G
c.844A>G
c.4451G>A
c.1666C>G
c.2246G>T
c.10477T>A
c.1605A>C
c.1465A>C
c.9445T>G
c.2398A>C
c.2728A>G
c.1965_1988del
c.2018A>G
c.371C>T
c.68C>A
c.1029G>C
c.307_318del
c.562G>T
c.1450C>G
c.2765G>A
[illegible]insCTGCTGGTTTTGGGAGTTCCCCAGCATTTGGAGCTGCAGCCTCTACCAGTTCAGGTATCTCTACTTCTGCTCCAGCTTTTGGATTT[illegible]

HGVSp	dbSNP_RS
p.Ser4079Thr	rs201606843
p.Ser1840Pro	rs780513634
p.Ile2164Val	novel
p.Arg759Trp	rs541845102
p.Asp1024Glu	rs777848041
p.Gln378Glu	novel
p.Lys379Glu	novel
p.Asn952Asp	rs775246786
p.Ser1697Pro	rs55659850
p.Lys2846Glu	rs151242026
p.Met3407Thr	rs201947164
p.Asp2086Glu	novel
p.Ile492Asn	rs796467869
p.Thr1230Pro	rs959635013
p.Met348Val	rs1785472
p.His66Tyr	rs747997151
p.Arg1664Ter	novel
p.Arg1568His	rs781253442
p.Val383Ala	rs9262394
p.His566Asp	rs577869411
p.Arg389Ile	
p.Arg436Gln	rs557613744
p.Val588Ile	rs377678499
p.Arg95Pro	
p.Arg377Cys	
p.Arg1337Gln	novel
p.Asn886Ser	rs776299242
p.Thr4080Pro	rs764364645
p.Ser617Asn	novel
p.Gly282Ser	novel
p.Arg1773Ser	rs200680705
p.Ser622Gly	rs773367375
p.Val1017_Ser1032del	novel
p.Gln1805His	novel
p.Leu433Ile	novel
p.Leu56Val	rs371459977
p.Thr3910Pro	
p.Arg286Cys	rs201107187
p.Phe593Ser	rs878968141
p.Ser536Gly	novel
p.Arg7Cys	rs774676700
p.Met328Ile	novel
p.Arg2956Ser	rs146122067
p.Ser1006Thr	novel
p.Thr2132Ala	rs34808481
p.Gln547Arg	novel
p.Glu620Ala	novel
p.Ala1185Thr	rs878937881
p.Ile1845Val	rs762913677
p.Leu424Pro	novel
p.Val1077Ala	rs866421353
p.Phe370Leu	novel
p.Ile3785Thr	rs984033598

p.Gly436Arg	rs766110702
p.Ser1856Pro	rs761889661
p.Ile3756Thr	novel
p.Ser573Arg	rs529292407
p.Lys440Ter	rs77618489
p.Ser3768Pro	rs999776161
p.Thr1161Pro	novel
p.Ser800Thr	
p.Ser237Gly	novel
p.Leu324Arg	rs782115581
p.Asn800His	rs541691706
p.Arg505Lys	rs879070793
p.Leu786Pro	rs199583642
p.Lys438Thr	rs782192834
p.Leu1280Pro	novel
p.Asn932Ser	rs878900949
p.Gly749Val	rs878925802
p.Ser269Ala	
p.Glu3301Asp	rs765465215
p.Ser713Phe	rs927887275
p.Leu2992Pro	rs75221587
p.Glu1432Gly	
p.Ser644Thr	rs551756814
p.Ser1813Gly	novel
p.Arg1071Ser	rs113038401
p.Glu694Asp	rs776073676
p.Asn857Asp	novel
p.Ala154Val	novel
p.Ala3786Thr	rs1019149594
p.Ala40Thr	rs200144130
p.Glu1718Lys	rs76484008
p.Ser3885Thr	novel
p.Arg158Cys	rs779838345
p.Glu925Asp	novel
p.Arg1389Gln	novel
p.Ser540Pro	novel
p.Val3769Ala	rs776435947
p.Cys223Arg	rs769247259
p.Ala648Val	
p.Glu473Lys	rs750997479
p.Arg1325Gln	rs1047094394
p.Arg1313Gln	novel
p.Ser1851Thr	rs777740569
p.Ile11Asn	novel
p.Ser360Arg	rs59612804
p.Leu1195Pro	rs781482765
p.Val278Ala	rs41318571
p.Asn4652Thr	novel
p.Ser1872Pro	rs200533147
p.Val188Ala	
p.Arg342Ile	rs374821944
p.Ile102Val	novel
p.Thr417Pro	rs767429863
p.Ser3705Ala	rs374442723

p.Asp1152Ala	rs533953825
p.Leu334Met	rs745796007
p.Met418Ile	
p.Phe353Ser	novel
p.Ile490Thr	rs749336598
p.Arg1330Cys	rs771294359
p.Asp3781Asn	rs403778
p.Gly188Ala	
p.Glu1336Gly	novel
p.Leu4134Pro	rs574341510
p.Asp1035Asn	rs199901845
p.Ala228Thr	novel
p.Trp1619Ter	
p.Cys509Arg	rs551242634
p.Lys470Asn	rs879035525
p.Phe593Leu	rs760218843
p.Met449Ile	rs746977407
p.Ala2302Thr	rs74542179
p.Leu464Pro	rs782743939
p.Glu3824Gly	novel
p.Leu1926Pro	novel
p.Ser517Pro	rs751516957
p.Ser505Pro	novel
p.Ile1085Thr	
p.Thr3958Pro	rs200317787
p.Asp494Gly	rs202081882
p.Thr1943Met	rs376988756
p.Cys265Arg	novel
p.Lys241Arg	novel
p.Asp3557Gly	novel
p.Cys583Arg	rs782482584
p.Ile3795Thr	novel
p.Gln2151Pro	rs745942889
p.Ser698Pro	rs202178544
p.Ser68Pro	novel
p.Arg88Gln	rs769657379
p.Gly747Ser	novel
p.Arg746Thr	novel
a1376_Arg1389delinsGl	novel
p.Arg1426Gly	novel
p.Leu3366Pro	rs879542121
p.Met1535Ile	rs200981553
p.Glu345Ter	rs76804900
p.Ala489Thr	rs370003523
p.Gly634Ser	rs80109605
elinsAspAlaGlnProGluSe	novel
p.Glu36AspfsTer53	novel
p.Ala169Thr	rs372244167
p.Pro187_Pro193del	rs765016382
p.Lys438Thr	rs782192834
p.Ile1206Thr	rs199901342
p.Ser647Leu	rs879117400
p.Ala235Ser	novel
p.Gly2306Asp	novel

p.Gln2057His	novel
p.Thr999Ala	novel
p.Ser541Thr	novel
p.Ser459Gly	rs924425209
p.Phe946Ser	rs746313861
p.Asn471Thr	rs754066869
p.Ser505Pro	novel
p.Ile1298Val	novel
p.Leu4735Ser	novel
p.Leu474Pro	novel
p.Glu658_Glu659del	rs149571560
p.Phe353Ser	novel
p.Ala381Pro	novel
p.Pro1041Ser	novel
p.Asn361His	novel
p.Thr404Ala	rs879169605
p.Ser1260Thr	
p.Ala4682Thr	rs962814170
p.Ser1813Gly	novel
p.Arg1805Ser	
p.Arg5347Gly	rs71449764
p.Arg1071Ser	rs113038401
p.Ser540Pro	novel
p.Gly634Ser	rs80109605
p.Val678Asp	novel
p.Ala498Thr	rs878861592
p.Pro238Gln	rs78071082
p.Trp4627Gly	rs61916081
p.Asp438Asn	rs776236647
p.Ser780Thr	
p.Ile5231Thr	rs879525667
p.Glu4298Gly	novel
p.Leu464Pro	rs782743939
p.Ala497Thr	rs201660744
p.Phe616Val	novel
p.Ile4718Val	novel
p.Val34Ala	rs202004345
p.Ser1077Gly	rs773326766
p.Glu416Lys	rs747838999
p.Met1178Val	novel
p.Arg308Ile	rs546238189
p.Met328Ile	novel
p.Thr2132Ala	rs34808481
p.Pro445Ser	novel
p.His383Leu	rs879955627
p.Glu5730Gly	novel
p.Ser476Gly	rs201369314
p.Phe856Ser	novel
p.Thr515Ala	novel
p.Arg1584Thr	novel
p.Ile114Phe	novel
p.Ala2169Val	rs201304037
p.Arg331Gln	rs753445925
Ile3785_Ile3795delinsT	novel

p.Thr2044Ala	novel
p.Arg1325Gln	rs1047094394
p.Asn593Ser	novel
p.Ser767Pro	rs140264686
p.Gly112Asp	rs370905698
p.Ile1131Thr	rs561412163
p.Phe698Ser	novel
p.Asp494Gly	rs202081882
p.Asn467Ile	novel
p.Val750Ile	rs760494369
p.Leu1195Pro	rs781482765
p.Arg3847Gly	novel
p.Gln2151Pro	rs745942889
p.Ile3846Thr	novel
p.Thr2510Ala	rs200591349
p.Arg167Thr	novel
p.Lys618Glu	rs557629431
p.Pro4224Ser	novel
p.Tyr492His	rs879950648
p.Asp1589Gly	rs771830770
p.Ile282Val	rs9262367
p.Arg1484Lys	rs468931
p.Gln556Glu	novel
p.Gly749Val	rs878925802
p.Ser3493Thr	novel
p.Gln535His	rs186425609
p.Lys489Gln	rs560526979
p.Ser3149Ala	rs533888763
p.Asn800His	rs541691706
p.Thr910Ala	
p.Glu658_Lys665del	
p.Glu673Gly	rs746551130
p.Pro124Leu	rs766412307
p.Ala23Asp	rs749262805
p.Leu343Phe	novel
p.Gly103_His106del	rs756495400
p.Val188Leu	
p.Leu484Val	novel
p.Ser922Asn	novel
p.Ala263CysfsTer163	novel
